# The evolving landscape of pharmacogenomics: Current achievements and future directions

**DOI:** 10.1016/j.pharmr.2026.100122

**Published:** 2026-02-02

**Authors:** Volker M. Lauschke, Magnus Ingelman-Sundberg

**Affiliations:** 1 Department of Physiology and Pharmacology, Karolinska Institutet, Stockholm, Sweden; 2 Center for Molecular Medicine, Karolinska Institutet, University Hospital, Stockholm, Sweden; 3 Dr Margarete Fischer-Bosch Institute of Clinical Pharmacology, Stuttgart, Germany; 4 University of Tübingen, Tübingen, Germany; 5 Department of Pharmacy, The Second Xiangya Hospital, Central South University, Changsha, China

## Abstract

Pharmacogenomics investigates how inherited and acquired genetic variation shapes drug efficacy, toxicity, and treatment failure. The major therapeutic areas for pharmacogenomics-assisted drug therapy include oncology, cardiology, psychiatry, neurology, infectious diseases, pain management, and metabolic disorders. Next-generation sequencing has revealed the extensive landscape of pharmacogenetic polymorphisms at the population scale. As a result, the field has evolved from early single-gene pharmacogenetics to genome-wide approaches that encompass the entirety of pharmacogenetic variability. However, much of the heritable variation in drug response remains unexplained, reflecting rare and structural variants, complex haplotypes, and the importance of polymorphisms in factors that regulate pharmacogenes in trans. A recently emphasized factor is the importance of considering differences in substrate specificities between enzymes and transporters that carry amino acid changes. Allele frequencies of actionable genetic variants are often low, requiring large, well powered studies that carefully account for key confounders, including patient adherence, placebo effects, comorbidities, hepatic and renal dysfunction, inflammation, and drug–drug and food-drug interactions. At the same time, emerging in silico variant-effect predictors, deep mutational scanning, population biobanks, and organotypic 3-dimensional human tissue models provide scalable platforms for functionally annotating variants and modeling human drug disposition and toxicity. A major effort moving forward is the continued identification and accurate classification of clinically important drug-gene pairs, along with improved implementation of pharmacogenomics in clinical practice. Artificial intelligence can accelerate this process by enabling rapid genome interpretation, prioritizing clinically relevant variants, and translating complex data into actionable recommendations. It can also integrate pharmacogenomic findings with other omics and help mitigate bias, thereby improving equity in treatment outcomes. In conclusion, the field will continue to expand, but its success will require large, rigorously designed ancestrally diverse trials, harmonized international regulatory standards, robust cost-effectiveness evidence, and the seamless integration of artificial intelligence-supported pharmacogenomic decision tools into global clinical practice.

**Significance Statement:**

Pharmacogenomics is a rapidly evolving field. Here, we review its foundational background, the most important clinical applications, and future perspectives with respect to methodological advances, the role of artificial intelligence, and its translation into clinical practice.

## I Short history of pharmacogenomics

The origins of pharmacogenetics can be traced back to the mid-20th century, when clinicians first noticed that some patients responded unusually well to certain drugs. For a thorough review of the history of pharmacogenomics, we refer the interested reader to an excellent previous review.^1^ Heritable sensitivity to primaquine in individuals with glucose-6-phosphate dehydrogenase (G6PD) deficiency, variable isoniazid metabolism (slow vs fast acetylators), and prolonged apnea associated with succinylcholine due to inherited pseudocholinesterase deficiency all suggested that genetic factors influenced drug response.^2^ This led to the birth of pharmacogenetics in the 1960s and 1970s, focusing on single-gene influences on drug metabolism and effects.^3^^,^^4^ Research on drug-metabolizing enzymes, especially cytochrome P450, has revealed profound interindividual and interethnic differences in enzyme activity.^5^^,^^6^ With the completion of the Human Genome Project (1990–2003), the scope expanded from single genes to genome-wide analyses, giving rise to pharmacogenomics—a broader field that considers multiple genes and pathways simultaneously.^7^^,^^8^ This shift was driven by high-throughput sequencing and genotyping technologies, which enabled the systematic identification of genetic variants that influence drug efficacy and toxicity. In the 2000s, pharmacogenomics entered clinical practice. Genetic testing has begun to guide treatment decisions; key examples include *HLA-B∗57:01* testing for abacavir hypersensitivity, CYP2C19 genotyping for clopidogrel response, and thiopurine S-methyltransferase (TPMT) testing for thiopurine dosing.^9^^,^^10^ Regulatory bodies such as the US Food and Drug Administration (FDA) now include pharmacogenomic biomarkers on drug labels, marking the integration of pharmacogenomics into modern personalized medicine.^11^

## II Evolutionary aspects

It is widely accepted that drug-metabolizing enzymes evolved through the long-standing biochemical arms race between plants and animals. Over evolutionary time, plants have developed increasingly complex biosynthetic pathways that produce diverse secondary metabolites—compounds essential for reproduction and defense against herbivores and pathogens. As animals incorporated plants into their diets, selective pressure favored plants that could synthesize toxic metabolites to deter consumption. In turn, animals evolved and diversified detoxification systems, including drug-metabolizing enzymes, to neutralize these ever-changing phytochemicals.^12^

Comparative genomic analyses revealed that major expansions in the *CYP1–CYP4* gene families coincided with the transition of animals from aquatic to terrestrial environments. This diversification was likely driven by dietary challenges, particularly exposure to plant-derived toxins encountered on land. Two complementary mechanisms appear to underlie species adaptation to environmental and dietary pressures. The first involves nuclear receptors that sense xenobiotics and regulate the expression of specific genes involved in their absorption, distribution, metabolism, and excretion (ADME), thereby providing a dynamic response that is conditional on chemical exposure. Notably, the structural similarities among xenobiotic-binding proteins, such as the pregnane X receptor, CYP3A4, and the efflux transporter P-gp (MDR1 and *ABCB1*), illustrate convergent evolution in their ability to recognize and respond to chemically diverse ligands.^13^ The second mechanism is based on genetic adaptation through the amplification of loci that encode enzymes involved in the detoxification of harmful compounds. In contrast to other biological responses, this evolutionary genetic adaptation is a slow process that typically occurs over thousands of years. Gene duplication is a well established mechanism driving molecular evolution, providing the raw material for the emergence of novel gene functions through neofunctionalization.^14^ However, recent evidence highlights the critical role of diet as a direct evolutionary driver of such amplification events, particularly in genes involved in xenobiotic detoxification.

### A Expansion of human genes

The first example of a functional human gene duplication was reported in 1993.^15^ The underlying mechanism involved unequal crossover at the *CYP2D* locus, where 3–4 genes with similar sequences are located, and alleles with 2, 3, 4, 5, and 13 copies of the *CYP2D6* gene were identified.^15^^,^^16^ Interestingly, the highest frequencies of *CYP2D6* gene duplications were first reported in Ethiopia^16^ and later in other parts of Northeast Africa.^17^ High frequencies have also been observed in North Africa and Arabia, with *CYP2D6* duplications occurring in 28% of individuals in Algeria and 10% in Saudi Arabia, as well as in southern Europe and Finland, where the prevalence ranges from 4% to 6%.^18^ Based on observations that certain toxic alkaloids display exceptionally high affinity for the CYP2D6 enzyme,^19^ it has been proposed that individuals with increased CYP2D6 activity may have had a selective evolutionary advantage.^20^

### B Diet-driven gene expansion in animals

**Fig. 1:**
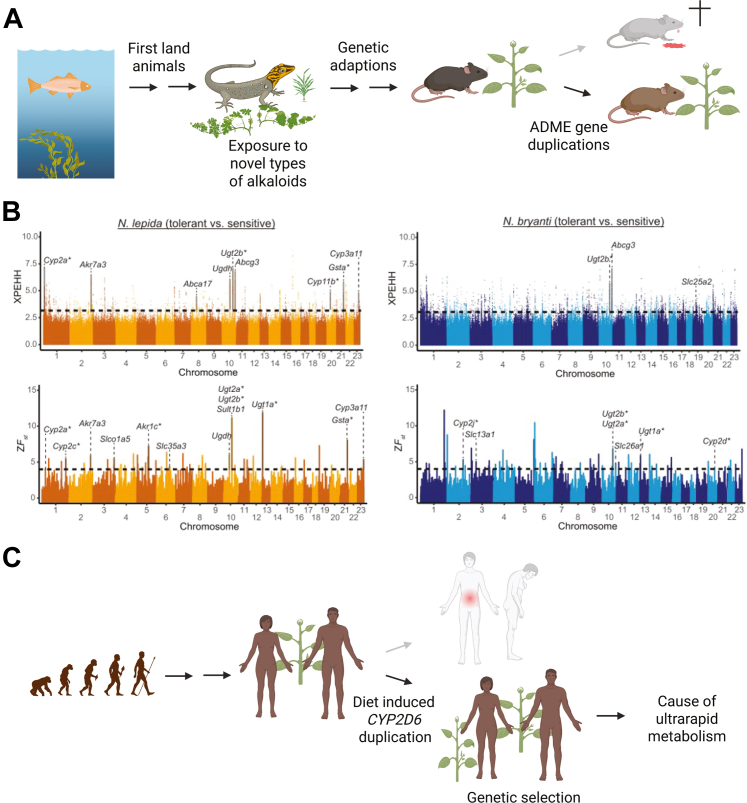
Diet-driven gene expansion. Gene duplication has been a major engine of evolutionary innovation, and growing evidence suggests that diet can serve as a key selective force shaping these genetic changes. (A) In woodrats, which have adapted to feed on the chemically defended creosote bush, toxic to most herbivores, survival is aided by duplications of multiple ADME-related genes, with *Ugt2b31* being the most prominent example. (B) Genomic regions that contain biotransformation-related genes, with signatures of recent positive selection in tolerant individuals among the *Neotomalepida* (in orange) and *Neotomabryanti* (in blue) strains, harbored multiple genomic regions with signatures of recent positive selection, as estimated from cross-population extended haplotype homozygosity (XPEHH: examines how an allele increased) and allele frequency differentiation (ZF_st_: examines where an allele increased) scans containing biotransformation genes, compared with conspecifics that were sensitive to creosote toxins (from Klure et al.^24^). (C) A parallel case occurs in humans, where dietary pressures in Northeastern Africa have promoted the duplication of the *CYP2D6* gene. This expansion broadens the diversity of consumable plants by enhancing CYP2D6-mediated detoxification of alkaloids. Figure adapted from Ingelman-Sundberg.^25^

This advantage may have enabled the respective carriers to tolerate a broader spectrum of plant-based toxins during periods of food scarcity, thereby exerting evolutionary pressure that favors gene amplification in these populations. Worldwide, similar selective pressures appear to have occurred not only in Northeast Africa but also in Oceania, involving duplication of the *CYP2D6∗1* variant allele.^17^ This case illustrates how diet-driven selection can influence modern pharmacogenomics, as carriers of *CYP2D6* duplications exhibit an ultrarapid metabolic phenotype that often requires dose adaptation across many different drugs in the clinical setting. In addition, *SULT1A1* has been shown to exhibit notable copy number variations (CNVs), with duplicated and multiduplicated alleles occurring at particularly high frequencies in African populations.^21^ The enzyme metabolizes several polyphenolic substrates, including apigenin, chrysin, epicatechin, quercetin, and resveratrol.^22^ Polyphenols are naturally occurring compounds present in a wide variety of plants.^23^ Although the current findings hint at possible functional or adaptive significance, no direct evolutionary explanation has yet been established. Interestingly, the paralog *Sult1b1* has been shown to undergo marked gene multiplication in woodrats that consume creosote-containing plant diets (Fig. 1),^24^^,^^25^ suggesting that sulfotransferase gene expansion can occur under strong dietary pressure.

Dietary-driven gene expansions have also been observed in the tobacco-adapted aphid *Myzus persicae nicotianae*, which acquired nicotine resistance through massive amplification (up to 100 copies) of *CYP6CY3*.^26^ This enzyme detoxifies nicotine and neonicotinoid insecticides better than human CYP2A6, illustrating how dietary toxins can drive both host-plant adaptation and pesticide resistance. A recent example has been described in woodrats (*Neotoma* spp.), where feeding on toxic creosote bushes independently evolved expansions of detoxification genes (*UGTs*, *GSTs*, *AKRs*, and P450s), with *Ugt2b31* copy number accounting for up to 78% of detoxification variation.^24^ Besides dietary selection, anthropogenic factors, such as the increasing use of pesticides, can contribute to the evolution of drug-metabolizing enzymes, particularly in insects.^27^, ^28^, ^29^ These examples demonstrate that dietary toxins repeatedly drive gene amplification, revealing diet as a potent and predictable architect of molecular evolution with lasting implications for medicine, agriculture, and conservation.

## III Overview of factors affecting interindividual differences in drug response and side effects

Pharmacogenomic variation contributes to interindividual differences in drug response and adverse effects, but it explains only about 20% of the variability observed in clinical practice. Other key determinants include drug–drug interactions, poor compliance, placebo effects, and renal and liver pathophysiology.^30^ Even among known genetic contributors, it is clear that many variants influencing drug metabolism and response remain unidentified. Behavioral factors and treatment adherence are often the most important—and most overlooked—determinants of drug response, explaining an estimated 30%–60% of unexplained variability.

Thus, according to the World Health Organization, up to 60% of patients with chronic diseases are nonadherent to therapy.^31^ In mental health and pain management, placebo effects also substantially influence outcomes. In clinical trials, eg, of novel selective serotonin reuptake inhibitors (SSRIs), up to 70%–75% of the apparent antidepressant effect was attributed to placebo responses.^32^ Similar placebo effects were observed in trials regarding the treatment of pain and anxiety.^33^ In clinical trials, it is of utmost importance to control for placebo effects using double-blinded trials; however, in open-label studies, such as the PREPARE trial, which aimed to validate the effect of ADME genes carrying actionable gene variants on adverse drug reactions, the placebo and other effects prevented reaching a conclusion.^34^, ^35^, ^36^

Drug–drug interactions represent a major source of variability that remains underappreciated in clinical guidelines.^37^^,^^38^ Strong CYP inhibitors can increase drug exposure (area under the curve [AUC]) by ≥5-fold, whereas strong inducers can reduce AUC by >80%. Interactions can also occur with dietary components. For instance, grapefruit juice strongly inhibits intestinal CYP3A4, increasing the AUC of CYP3A4 substrates, such as simvastatin and buspirone, by 2–4-fold and up to 10-fold under high-intake conditions.^39^^,^^40^ Smoking induces CYP1A2, resulting in reduced plasma concentrations of substrates such as clozapine by 30%–50%.^41^

Kidney and liver function play critical roles in drug clearance. For renally eliminated drugs, clearance typically decreases proportionally to the glomerular filtration rate.^42^ Hepatic impairment can similarly reduce metabolism, increasing systemic exposure 2–5-fold, depending on the pathway involved.^43^ Finally, local or systemic inflammation can downregulate hepatic drug metabolism. Proinflammatory cytokines, especially interleukin-6, suppress various CYP enzymes,^44^ and in conditions such as rheumatoid arthritis, CYP3A4 activity may decrease by 50%–99%, substantially increasing exposure to affected drugs.^45^

In conclusion, it is important to consider genetic and nongenetic factors in concert in all clinical pharmacogenomic studies to avoid potential sources of bias. Furthermore, it is critical that such studies be conducted using single-blind or, preferably, double-blind designs to minimize placebo effects and nonpharmacological confounders.

## IV Missing heritability

### A General aspects

Heritable variability in pharmacogenomic traits can be broadly categorized into 3 groups: (1) monogenic (Mendelian) traits, which are typically driven by 1 or a few genetic variants; (2) oligogenic traits, largely determined by a small number of major pharmacogenes; and (3) complex pharmacogenomic traits caused by a single or combined effects of innumerable small-effect genetic variants.^46^ A major limitation of current pharmacogenomic research is the incomplete understanding of the genetic influence on interindividual differences in drug metabolism, efficacy, and toxicity. Twin studies suggest that a substantial proportion of this variability remains unexplained, highlighting the complexity of the underlying genetic architecture.^47^

In contrast, the genetic basis of a well studied polygenic trait, such as height, has recently been comprehensively characterized. A landmark genome-wide association study (GWAS) of 5.4 million individuals from diverse ancestries identified 12,111 independent single-nucleotide variants (SNVs) that reached genome-wide significance for human height. Together, these variants account for nearly all of the SNV-based heritability attributable to common genetic variation.^48^ These variants are distributed across 7209 nonoverlapping genomic segments, with a mean size of approximately 90 kb, covering about 21% of the genome.

Although a similar scale of association is not expected for the ADME genes, the extent of genetic variation within these loci is nevertheless substantial. An integrative analysis of more than 5000 Chinese individuals, encompassing 604 ADME genes, identified approximately 33,925 unique SNVs and 484 indels across multiple sources, based on whole-exome sequencing.^49^

Beyond coding variants, numerous polymorphisms in regulatory regions also contribute to the genetic diversity of ADME genes. Combinations of these variants generate a vast number of distinct haplotypes, many of which, such as those within the *CYP2D* locus, are associated with interindividual differences in drug-metabolism phenotypes.^50^ A major limitation to the potential clinical application of such haplotypes is the pronounced interethnic differences in their distribution and associated functional effects. For example, a haplotype within the *CYP2C* locus that contains genetic variants in *CYP2C18* (designated *CYP2C:TG*) has been identified as a major haplotype associated with markedly increased CYP2C19-mediated drug metabolism, with a frequency of approximately 19% in Norway.^51^ Individuals carrying this haplotype exhibit significantly higher metabolic rates for drugs such as escitalopram compared with those harboring the reference allele *CYP2C19∗1*, and even slightly higher activity than carriers of the ultrarapid metabolizer (UM) allele *CYP2C19∗17*. In several indigenous American populations and in South American cohorts, the same *CYP2C:TG* haplotype is even more common, with frequencies ranging from 47% to 60%,^52^ and recent studies in Native Mexican populations indicate increased activity based on the metabolic ratio of omeprazole.^53^

However, this relationship does not appear to be consistent across populations. For instance, increased metabolism was not observed in a pediatric cohort of 41 children prescribed proton pump inhibitors, although the relatively small sample size may also have contributed to the lack of observed effects.^54^ Similarly, no effects were observed in a study of 222 healthy volunteers receiving diverse CYP2C19 substrates, which, however, did not include escitalopram.^55^ This heterogeneity makes direct comparisons challenging. These findings illustrate that, without a clearly defined functionally causal variant, genotype–phenotype extrapolations based solely on haplotype differences remain inherently uncertain.

Furthermore, recent studies indicate that single SNVs across a broader region of the *CYP2D* locus contribute to interindividual variation in CYP2D6-dependent drug metabolism.^50^^,^^56^ Furthermore, variability in CYP2D6 can be associated with genetic variation in *NFIB,* as discussed in detail below. With respect to variation in tamoxifen metabolism, cross-ancestry genome-wide data from 497 patients of European, Middle Eastern, and Asian descent showed that all genetic variants relevant to interindividual differences in CYP2D6-dependent metabolism are located on chromosome 22, in proximity to the *CYP2D* locus.^56^ However, it is not yet plausible to incorporate these genetic variations into algorithms for predicting CYP2D6 activity until their exact functional roles are better understood.

Taken together, although numerous genetic variants influencing drug response have been identified, they collectively account for only a fraction of the observed heritability. This gap limits our understanding of interindividual variability in drug efficacy and toxicity, thereby constraining the advancement of truly precise and personalized therapies. Contributing factors to this missing heritability include rare and structural variants, complex gene-environment interactions, and incomplete haplotype characterization. Because conventional GWAS predominantly focus on common SNVs, much of the broader genetic architecture underlying pharmacogenomic traits remains unexplored. To overcome these limitations, integrative approaches that combine whole-genome sequencing, multiomics analyses, and machine-learning-based modeling of rare variants and nongenetic influences are needed. Furthermore, inclusion of diverse populations is essential to minimize bias and enhance the global applicability of pharmacogenomic discoveries.

### B Endogenous/dietary biomarkers

One approach to overcoming missing heritability and other determinants of interindividual variability is to use endogenous or dietary biomarkers, in which the ratio of substrate to metabolite concentrations reflects the metabolic phenotype. Significant progress is being made in this area, and the most successful example has been the identification of CYP2D6 poor metabolizers (PMs) with >95% specificity by measuring the ratio of solanidine, a dietary steroidal alkaloid from potatoes, to its metabolite, 4-OH-solanidine, using mass spectrometry.^57^^,^^58^ Unlike traditional CYP2D6 probe drugs, this method requires no drug administration, making it noninvasive and well suited for large-scale phenotyping. However, solanidine metabolic ratios cannot discriminate between intermediate metabolizers (IMs), normal metabolizers (NMs), and UMs.

### C The polymorphic nuclear factor IB

One factor contributing to the missing heritability is nuclear factor (NF)IB, a member of the NFI transcription factor family that also includes NFIA, NFIC, and NFIX. In a GWAS of clozapine-treated patients, the *NFIB* variant rs1923778 T>C was associated with symptom severity (*P* = 3.78 × 10^-7^).^59^ In a later study, it was found that NFIB modulates CYP2D6 activity; among CYP2D6 NMs, *NFIB* rs28379954 T>C allele carriers showed substantially higher 9-hydroxyrisperidone/risperidone metabolic ratios, effectively shifting their metabolism toward an UM-like phenotype, whereas no effect was observed in CYP2D6 PMs.^60^ In the human liver, NFIB is nuclear, and individuals carrying the rs28379954 C variant allele exhibited reduced hepatic NFIB expression. NFIB overexpression markedly suppressed CYP2D6 promoter activity in Huh7 cells, and a regulatory element was identified upstream of the transcription start site. Increased CYP2D6 expression in relation to the *NFIB* C variant was explained by reduced inhibition due to lower NFIB expression.^60^ Similar findings were observed with solanidine as a substrate, where the effect of NFIB suggested a 200% increase in CYP2D6-mediated clearance in NMs heterozygous or homozygous for NFIB rs28379954 T>C.^61^ Data furthermore suggest that NFIB acts as a repressor of key efflux transporters in the intestinal epithelium. *In vitro*, NFIB represses *ABCB1* and *ABCG2* expression. In a cohort of 285 clozapine-treated patients, rs28379954 C-allele carriers with lower NFIB expression exhibited lower systemic clozapine levels, consistent with increased efflux of clozapine back into the gut lumen.^62^ Hence, for clozapine, the primary NFIB-linked effect appears to be on transporters rather than on metabolizing enzymes, as CYP enzymes involved in clozapine metabolism did not show clear genotype-dependent activity changes. Notably, no effects of rs28379954 were observed in other studies on propafenone hydroxylation or on the metabolic ratios of tamoxifen, sparteine, and solanidine.^63^^,^^64^ The reasons for these discrepancies need to be investigated.

A recent study examined the roles of NFIB family members in regulating ADME genes in primary 2-dimensional hepatocytes and found that knockdown of NFIB, NFIC, and NFIX resulted in broad suppression of CYP drug-metabolism pathways.^63^ This suggests that multiple CYP enzymes, including those in the CYP3A and CYP2C families, may be under NFI transcriptional control. At present, CYP2D6 is the only CYP enzyme with robust, replicated evidence linking *NFIB* genetic variation to functional consequences in humans, as demonstrated using risperidone and solanidine substrates in independent cohorts.^60^^,^^61^ For other CYP enzymes, NFIB is mechanistically implicated by expression studies, but no firmly established CYP-specific pharmacogenetic effect of NFIB has been demonstrated in vivo.

## V Substrate specificity

Pharmacogenomic variability primarily arises from genetic variants that alter gene function or expression. Most of these variants are missense variants that cause amino acid substitutions, frameshift or nonsense mutations that lead to altered enzyme activity, or regulatory-region variants that affect transcription and gene expression levels. Such mutations in drug-metabolizing enzymes and transporters pose a particular challenge because their functional impact often depends on the substrate involved.

Among the key ADME genes, several clinically relevant examples illustrate this substrate-dependent behavior.

CYP2B6.4: The p.K262R variant causes distinct specificities for different substrates, such as bupropion and benzphetamine.^65^

CYP2C8.3: The p.R139K and p.K399R variants show substrate-dependent differences in activity, with increased metabolism of paclitaxel and repaglinide but reduced activity toward amodiaquine.^66^

CYP2D6.2, CYP2D6.17, CYP2D6.29, and CYP2D6.35: These allelic variants are associated with altered enzyme activity, with substrate-specific effects documented across multiple studies.^50^^,^^67^

ABCG2.2 (BCRP): The p.Q141K variant reduces protein stability and transport capacity, resulting in substrate-specific pharmacokinetic changes across various drugs.^68^

In most cases, current pharmacogenetic guidelines do not adequately account for substrate-specific differences. For example, for substrates such as tamoxifen and risperidone, the activity scores assigned to *CYP2D6* variants are substrate-agnostic and do not account for differences in substrate metabolism. Consequently, recommendations from the Clinical Pharmacogenetics Implementation Consortium (CPIC) for predicting in vivo metabolic activity may be unreliable for these drugs. However, when activity scores are modified to reflect the actual substrate specificity of CYP2D6 variants, substantially improved prediction accuracy is achieved.^60^^,^^67^

Given that these variants are common and their allele frequencies vary widely across global populations, there is a pressing need to systematically define and validate substrate-specific activity scores for individual drug-gene variant pairs.

## VI Types of pharmacogenomic variants and rearrangements

**Fig. 2:**
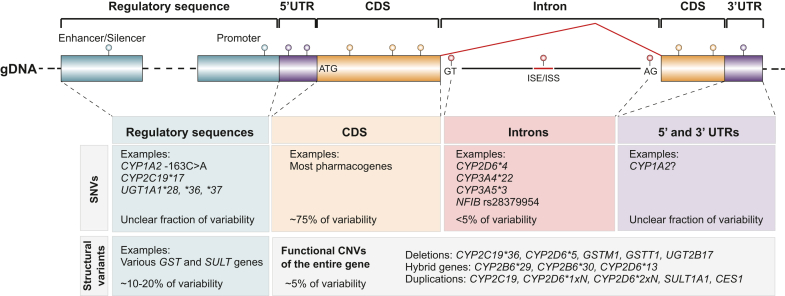
The landscape of pharmacogenomic variability. Variants can be classified into point mutations affecting 1 or a few bases (indels), collectively referred to as SNVs, and structural variants, defined as rearrangements affecting >50 bp. SNVs with demonstrated functional effects can be found in regulatory sequences, coding sequences (CDS), and intronic regions, including splice donor and acceptor sites, as well as intronic splicing enhancer/silencer (ISE/ISS) regions. Evidence of functionally relevant variants in the UTRs of pharmacogenes is currently limited. Structural variants can affect functional elements in regulatory regions or impact the gene body via full or partial duplication or deletion. Furthermore, multiple pharmacogenes undergo complex rearrangements, resulting in mostly inactive hybrids with nearby pseudogenes.

Pharmacogenomic variability comprises different types of variations (Fig. 2). Among these, SNVs are the most prevalent. This type of variation can be found in coding or noncoding regions. Coding SNVs have been most extensively investigated, and missense and nonsense variants in virtually all pharmacogenes have been associated with functional consequences. In addition, there is an increasing number of synonymous variants that affect gene function through different mechanisms. Examples include in p.Ile1145Ile (rs1045642) in *ABCB1*,^69^ which alters translational efficiency and kinetics; p.Pro319Pro (rs6277) in the dopamine receptor *DRD2*,^70^ which reduces mRNA stability; and p.Pro227Pro (rs4244285) in *CYP2C19*, which disrupts splicing.^71^ SNVs have also been reported in regulatory sequences, as is the case for *CYP2C19∗17* (rs12248560) and the -163C>A (rs762551) polymorphism in *CYP1A2*, as well as in untranslated regions (UTRs), which can impact transcript levels and miRNA regulation.^72^ However, although 3%–4% of genetic associations for quantitative traits map to UTR variants,^73^ and a few examples of UTR variants in CYP1A2, CYP2D6, and CYP3A4 with possible effects have been described,^74^ their overall functional impacts on pharmacogenomic phenotypes remain unclear.

Besides SNVs, small insertions and deletions represent another class of pharmacogenomic variants. The arguably most prominent examples are *DPYD∗3* (rs72549303), a 1-bp deletion that results in a frameshift and a loss-of-function gene product, and indel polymorphisms in the TATA box element of the *UGT1A1* promoter (*UGT1A1∗28*, *∗36,* and *∗37*) that determine UGT1A1 expression.

In addition to these small variants, pharmacogenes can be subject to large structural rearrangements, such as deletions, duplications, and inversions, that affect ≥50 bp of DNA. Notably, while the number of such structural variations in the human genome is around 2 orders of magnitude lower than that of SNVs (34,000 structural variants vs 3 million SNVs), structural rearrangements affect 3.4 times more nucleotides.^75^ Such complex events have been difficult to resolve using short-read sequencing but are increasingly detected with long-read technologies, which enable accurate reconstruction of structurally complex pharmacogenes.^76^^,^^77^ In pharmacogenetics, CNVs are extensively studied in *CYP2D6,* where structural variants can give rise to gene deletions, duplications/multiplications, and hybrid genes.^78^^,^^79^ However, other pharmacogenes, such as *CYP2A6*, *GSTM1*, *GSTT1*, *SULT1A1,* and *UGT2B17*, also carry common CNVs.^80^ These variations can be highly overrepresented in certain populations, as evidenced by putatively deleterious partial exon 4 deletions of *DPYD*, which have been identified in 31% of patients carrying a pathogenic DPYD variant in the Finnish population.^81^ Moreover, recent work based on population-scale sequencing data identified exonic deletions and duplications in 97% of the 208 analyzed pharmacogenes.^82^ These structural variants accounted for >5% of all loss-of-function alleles in almost half of the analyzed genes. In addition, analyses of whole-genome sequencing data revealed noncoding structural variants within pharmacogenomic loci, ranging in size up to 106 Mb and commonly overlapping gene regulatory elements.^83^ A further new domain area is structural variability in intronic regions. Intronic CNVs can alter gene expression levels and have been described, for instance, for *ABCC1* and *CYP4F11*.^84^ However, their overall functional impact in the context of pharmacogenomics has not yet been evaluated.

Based on these results, we estimate that 75% of the known genetically encoded pharmacogenomic variability is attributable to SNVs and indels, whereas noncoding and coding structural variants account for 20% and 5%, respectively.

## VII Pharmacogenomics across therapeutic areas: Current clinical biomarkers and their significance

**Fig. 3:**
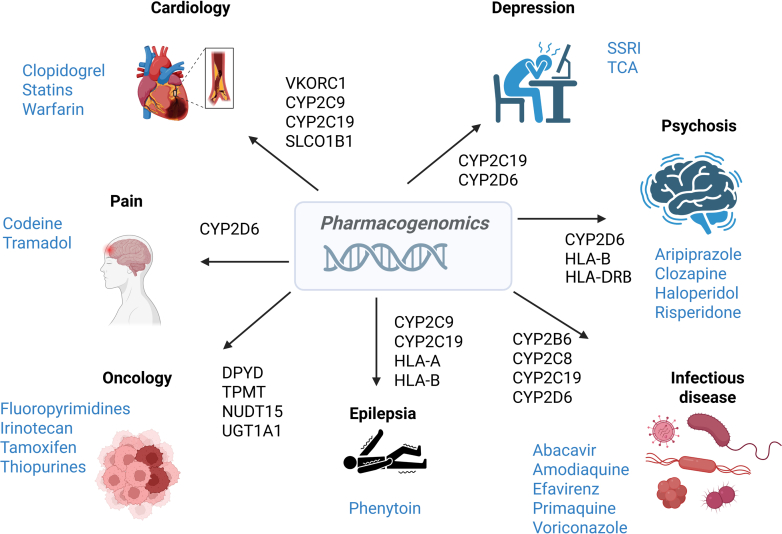
The currently most important clinical applications of pharmacogenomics. The figure highlights the key pharmacogenomic biomarkers associated with the indicated genes across various therapeutic areas. Incorporating these biomarkers into clinical decision-making can enhance drug efficacy and reduce the incidence of adverse drug reactions. Hereby, more personalized and safer treatment strategies are obtained. TCA, tricyclic antidepressant.

Pharmacogenomics plays a critical role across several clinical fields, including oncology, psychiatry, neurology, and cardiovascular medicine (Fig. 3). In oncology, the primary clinical focus has traditionally been on somatic genetic variants, which guide the selection and dosing of anticancer therapies. The implementation and interpretation of somatic testing are largely performed by pathologists and oncologists, as these variants arise within the tumor and directly inform targeted treatment strategies. In contrast, germline pharmacogenomic variants, which influence drug metabolism, toxicity risk, and overall treatment tolerability across all medical specialties, fall more commonly within the scope of pharmacists, clinical pharmacologists, and general practitioners. Although germline variants are also relevant in oncology, especially for predicting adverse drug reactions or inherited cancer risk, they are often underused compared with somatic markers. As precision medicine continues to evolve, integrating both somatic and germline pharmacogenomic information will become increasingly important for optimizing therapeutic outcomes and minimizing toxicity across oncology and other medical disciplines.

Pharmacogenomic information in product labels provides a clinically useful framework for predicting drug treatment efficacy and safety. Such labeling is issued by agencies including the FDA, the European Medicines Agency (EMA), CPIC, and the Dutch Pharmacogenetics Working Group (DPWG); however, the definitions, classifications, and practical use of pharmacogenomic labels vary substantially across organizations.^85^ The FDA maintains a public Table of Pharmacogenomic Biomarkers in Drug Labeling,^11^ which lists drugs containing pharmacogenomics (PGx) information from the Drugs@FDA database. This resource currently includes more than 260 drugs and over 360 drug-biomarker pairs across therapeutic areas, including oncology (representing ∼50% of all labels), neurology, infectious diseases, pain/rheumatology, and psychiatry. Not all entries require genetic testing; rather, only 29% recommend or mandate testing, most commonly in oncology, where toxicity risk or targeted therapy selection is critical.

The FDA also provides a complementary Table of Pharmacogenetic Associations,^86^ which categorizes gene-drug pairs into 3 groups: (1) evidence-based therapeutic PGx associations, (2) PGx associations with potential impact on safety or response, and (3) PGx associations that affect pharmacokinetics only.

Section 1 of this FDA resource includes 60 gene-drug pairs for which evidence supports therapeutic management recommendations (Table 1). Of these, 45 involve CYP genes, with *CYP2D6* occurring most frequently, featured in 25 clinically significant gene-drug associations. As is evident, the highest number of drug-gene pairs is in the therapeutic areas of neurology, oncology, and psychiatry. This stratification approach highlights the clinical relevance of pharmacogenomic variants; however, as new evidence emerges, these classifications require continual reassessment and validation. However, important differences remain in how the FDA, EMA, CPIC, and DPWG define and classify pharmacogenomic information. Establishing a global, harmonized standardization system that explicitly accounts for interethnic variation would provide substantial added value.

**Table 1:** Gene-drug pairs with therapeutic management recommendations according to the FDA; data were accessed in December 2025

Category	Drug	Gene(s)
Anesthesiology	Mivacurium	*BCHE*
	Succinylcholine	*BCHE*
Cardiology/hematology	Clopidogrel	*CYP2C19*
	Propafenone	*CYP2D6*
	Warfarin	*CYP2C9, CYP4F2, VKORC1*
Dermatology/immunology	Abrocitinib	*CYP2C19*
Endocrinology/metabolic Disorders	Eliglustat	*CYP2D6*
	Nateglinide	*CYP2C9*
Gastroenterology	Metoclopramide	*CYP2D6*
	Pantoprazole	*CYP2C19*
Infectious diseases	Abacavir	*HLA-B*
Immunology/transplantation	Azathioprine	*TPMT, NUDT15*
	Mercaptopurine	*TPMT, NUDT15*
	Tacrolimus	*CYP3A5*
	Thioguanine	*TPMT, NUDT15*
Neurology	Amifampridine	*NAT2*
	Brivaracetam	*CYP2C19*
	Carbamazepine	*HLA-B*
	Clobazam	*CYP2C19*
	Fosphenytoin	*CYP2C9, HLA-B*
	Meclizine	*CYP2D6*
	Phenytoin	*CYP2C9, HLA-B*
	Pitolisant	*CYP2D6*
	Tetrabenazine	*CYP2D6*
	Valbenazine	*CYP2D6*
Oncology	Belinostat	*UGT1A1*
	Belzutifan	*CYP2C19, UGT2B17*
	Capecitabine	*DPYD*
	Erdafitinib	*CYP2C9*
	Fluorouracil	*DPYD*
	Gefitinib	*CYP2D6*
	Irinotecan	*UGT1A1*
	Mercaptopurine	*TPMT, NUDT15*
	Sacituzumab	*UGT1A1*
	Thioguanine	*TPMT, NUDT15*
Pain/rheumatology	Celecoxib	*CYP2C9*
	Codeine	*CYP2D6*
	Flurbiprofen	*CYP2C9*
	Meloxicam	*CYP2C9*
	Oliceridine	*CYP2D6*
	Piroxicam	*CYP2C9*
	Tramadol	*CYP2D6*
Psychiatry	Amphetamine	*CYP2D6*
	Aripiprazole	*CYP2D6*
	Atomoxetine	*CYP2D6*
	Brexpiprazole	*CYP2D6*
	Citalopram	*CYP2C19*
	Clozapine	*CYP2D6*
	Deutetrabenazine	*CYP2D6*
	Flibanserin	*CYP2C19*
	Iloperidone	*CYP2D6*
	Lofexidine	*CYP2D6*
	Pimozide	*CYP2D6*
	Thioridazine	*CYP2D6*
	Venlafaxine	*CYP2D6*
	Vortioxetine	*CYP2D6*

### A Psychiatry

#### 1 Depression

Major depressive disorder (MDD) affects over 280 million people worldwide and is a leading cause of disability. Antidepressants (primarily SSRIs, serotonin and norepinephrine reuptake inhibitors, and tricyclic antidepressants) are first-line treatments, yet only about 40% of patients achieve remission with the initial medication, leading to a prolonged trial-and-error process.^87^^,^^88^ This heterogeneity is partly attributable to genetic differences that affect drug metabolism, transport, and pharmacodynamic targets. CYP enzymes are the most studied targets in antidepressant pharmacogenomics, particularly CYP2D6 and CYP2C19, whose polymorphisms affect the plasma levels of SSRIs, tricyclic antidepressants, and some serotonin and norepinephrine reuptake inhibitors. Particularly important are polymorphisms in *CYP2C19*: the common allele *CYP2C19∗2* is a missense loss-of-function variant, and *CYP2C19∗17* is an UM allele. In addition, a haplotype variant carrying *CYP2C19∗1*, referred to as *CYP2C:TG*, has been shown to cause an increase in the metabolism of sertraline,^89^ escitalopram,^51^ and the proton pump inhibitor omeprazole.^90^ However, as mentioned, the mutations defining this haplotype are not in complete linkage with the genetic variants that cause altered activity, and the *CYP2C:TG* haplotype is not sufficient for classification as a UM variant.

Evidence supporting PGx-guided antidepressant selection has strengthened over the last few years. A 2024 position statement from the American Psychiatric Association highlights that multiple randomized controlled trials now show moderate benefits in response and remission rates for patients with treatment-resistant or difficult-to-treat depression when combinatorial PGx testing informs prescribing, with the strongest effects in those who have failed prior treatments.^91^ Long-term analyses of trials, including the GUIDED study, demonstrate that benefits in remission persist through 24 weeks and are associated with reduced healthcare utilization.^92^^,^^93^ An important study by the University of British Columbia developed a simulation model to evaluate the cost-effectiveness of pharmacogenomic-guided treatment for MDD in British Columbia.^94^ They found that 37% fewer patients experienced treatment-resistant depression when pre-emptive genotyping was used. The model predicted that implementing pharmacogenomic testing for 194,149 adult patients could save the health system approximately $956 million over 20 years, while also yielding health gains of 12,436 life-years and 74,023 quality-adjusted life-years.

Several meta-analyses have been published based on large cohorts. For instance, an integrative analysis of 13 trials involving a total of 4767 patients found that patients receiving pharmacogenetically guided antidepressant therapy were 40% more likely to achieve remission than those in the treatment-as-usual group.^95^ A similar increase in remission was described in other meta-analyses that analyzed studies using a weighted multigene pharmacogenomic test for adults with depression.^96^, ^97^, ^98^

Real-world effectiveness has been further validated in large cohort studies. A 2025 US insurance claims analysis of over 10,000 MDD patients showed that pharmacogenetically tested individuals had significantly lower rates of psychiatric hospitalization, emergency visits, and antidepressant switches than untested controls, resulting in both clinical improvement and cost savings.^99^ Additional observational data reinforce that switching to genotype-congruent medications markedly improves outcomes in prior nonresponders.^100^

CPIC provides regularly updated, evidence-based dosing guidelines for most antidepressants according to CYP2D6 and CYP2C19 phenotypes.^101^ The FDA tables include PGx information in labels for over 40 antidepressants, and insurance coverage for testing in nonresponders is expanding.

#### 2 Schizophrenia and psychosis

Pharmacogenomic influences on treatment outcomes in schizophrenia and psychosis have generally been found to be modest. The current body of evidence either supports pharmacogenetics-guided prescribing or indicates that there are no significant differences between pharmacogenetic approaches and treatment as usual with respect to both clinical and economic outcomes.^102^ Similarly, a large synthesis of 29 meta-analyses, based on pooled data from 298 original studies and more than 69 pharmacogenetic variants across 39 genes, reported small overall pharmacogenomic effects on antipsychotic treatment response and weight gain.^103^

More convincing evidence for a role of pharmacogenomics has emerged with respect to drug exposure and metabolism of antipsychotic medications, also connected to drug switching. A meta-analysis comprising 94 studies and 8379 patients identified significant effects of the CYP2D6 phenotype on serum levels of aripiprazole, haloperidol, and risperidone.^104^ For risperidone in particular, UMs and PMs were shown to markedly affect plasma drug concentrations as well as treatment switching, with both groups demonstrating substantially higher switching rates.^105^ The retrospective genotyping of patients revealed a pronounced phenotype-dependent effect on risperidone exposure.

Treatment with clozapine is influenced by genetically determined pharmacokinetics as well as susceptibility to adverse drug reactions. Clozapine metabolism is affected by the -163C>A (rs762551) polymorphism (previously *CYP1A2∗1F*), although the overall magnitude of this effect is relatively modest. A meta-analysis of clozapine and norclozapine blood levels, incorporating 3 independent GWAS datasets comprising a total of 6802 patients, demonstrated that clozapine concentration-to-dose ratios were significantly associated with rs2472297, located between *CYP1A1* and *CYP1A2*, and with *UGT2B10* rs835309.^106^ Interestingly, Solbakk et al^62^ provided evidence that NFIB-dependent polymorphic regulation of the *ABCG2* transporter influences circulating clozapine concentrations independently of metabolic pathways.

The most serious complication of clozapine therapy remains the risk of clozapine-induced agranulocytosis (CIA), which can be fatal. Based on a recent review of pharmacogenomic studies addressing CIA risk, it was concluded that individuals carrying the *HLA-DRB1∗04:02* allele had nearly a 6-fold increase in the odds of developing CIA compared with noncarriers.^107^ In addition, several other variants in *HLA-DQB1*, *HLA-B∗38*, *HLA-B∗59,* and *HLA-DR4*, have also been implicated. *HLA-DRB1∗04:02* is considered the primary risk allele for CIA; furthermore, *HLA-B∗59:01* is a population-specific risk allele for testing in Japan.^108^ However, data regarding polymorphic clozapine effects need further replication before they can be implemented in clinical psychiatry.

### B Neurology

Patients often respond differently to the same antiseizure medication, and the therapeutic window for many of these drugs is narrow. Genetic information is therefore valuable for predicting adverse effects, particularly immunologically mediated reactions such as Stevens-Johnson syndrome and toxic epidermal necrolysis. In addition, polymorphisms in genes encoding drug-metabolizing enzymes can substantially influence drug levels and contribute to variability in treatment response.

While genetic variability has shown only limited power in predicting antiseizure drug resistance or overall treatment response in large cohort studies examining drug targets (eg, *SCN1A*) and transporters such as *ABCB1* and *ABCC2*,^109^, ^110^, ^111^ the situation is different for adverse drug reactions. Polymorphic variants of specific *HLA* alleles, such as *HLA-B15:02, HLA-A∗31:01*, *HLA-B∗15:11,* and *HLA-B∗15:21*, have consistently demonstrated strong associations with severe cutaneous adverse reactions in patients receiving certain antiseizure medications.^112^, ^113^, ^114^, ^115^, ^116^ Thus, the relationship between *HLA* polymorphisms and the risk of cutaneous adverse drug reactions remains the most clinically important application of pharmacogenomics in epilepsy care,^117^ and evidence has also been presented for the cost-effectiveness of genotyping prior to treatment of these *HLA* alleles.^118^

Weaker associations have been observed in the field of pharmacokinetics. A large meta-analysis of 98 studies involving 12,543 adult participants found that among various antiseizure medications, only phenytoin showed a significant effect related to *CYP2C9* and *CYP2C19* polymorphisms.^119^ By contrast, research on the influence of drug transporters and ion channel variants has not produced substantial or clinically meaningful findings in this area.

### C Metabolic disease

Pharmacogenomics has impacted the therapy and management of multiple conditions within the metabolic disease spectrum. For type 2 diabetes mellitus (T2DM), the arguably most extensively studied agent is metformin, a first-line therapeutic for the management of T2DM that lowers blood sugar levels by suppressing hepatic gluconeogenesis; however, the drug also has pleiotropic effects on various other organ systems and metabolic processes.^120^ Metformin pharmacokinetics and response are subject to interindividual differences, with pharmacogenetic variants, mostly in transporter genes, repeatedly implicated but inconsistently replicated.^121^^,^^122^ Heritability estimates based on twin studies^123^ or a 6-generation population-sized pedigree from national registries comprising 80,788 T2DM patients treated with metformin^124^ suggested that metformin responses are predominantly regulated by nongenetic factors.

The situation is similarly controversial for sulfonylureas. Many are metabolized by CYP2C9, and the reduced-function alleles *CYP2C9∗2* and *CYP2C9∗*3 have been linked to decreased hepatic clearance^125^, ^126^, ^127^ and increased therapeutic efficacy.^128^, ^129^, ^130^ However, safety associations remain unclear. A large multiethnic study of 71,857 individuals, including 4056 sulfonylurea-treated patients, found that *CYP2C9∗3* was associated with a cardiac safety risk in admixed/Latino individuals (*P* = 2 × 10^-4^) but not in Europeans (*P* = .42),^131^ possibly due to lower allele frequencies in Europe and the Middle East.^132^ In Scotland, *CYP2C9* reduced-function variants were associated with hypoglycemia (odds ratio [OR], 2.8; *P* = .009) in 1770 users,^133^ whereas a similarly sized US cohort found no association with genotype.^134^ Consequently, pharmacogenetic guidelines do not currently recommend sulfonylurea dose adjustments based on *CYP2C9* genotype.^135^

Incretin pathway drugs targeting glucagon-like peptide-1 (GLP-1) and glucose-dependent insulinotropic polypeptide (GIP) signaling have become highly effective for treating T2DM, obesity, and metabolic dysfunction-associated steatotic liver disease. Since exenatide was approved in 2005, GLP-1 receptor agonists (GLP-1RAs) have also demonstrated cardiovascular and renal benefits.^136^ Furthermore, they are being explored for type 1 diabetes, Alzheimer disease, and sleep apnea.^137^ Drug development initially emphasized half-life extension but now focuses on a shift from injectable peptide drugs toward orally available small molecules. These trends are paralleled by a shift from mono-agonism of the GLP-1 receptor to dual/triple agonists that also target the GIP and glucagon receptors.^138^^,^^139^ Notably, both GIP receptor (GIPR) agonists and antagonists appear to enhance weight loss,^140^, ^141^, ^142^ raising questions about whether GIP stimulation or inhibition is more beneficial. Human genetic studies show that reduced-function GLP-1R variants are associated with impaired glycemic control and higher adiposity,^143^ whereas GIPR variants show the opposite pattern.^144^ These results are consistent with the hypothesis that long-term GIPR agonism may lead to desensitization, resulting in effects similar to those observed with GIPR antagonist treatment.^145^

Pharmacogenetic studies of incretin-modulator responses are limited. The GLP-1R variants rs10305420 and rs6923761 were linked to improved GLP-1RA efficacy,^146^^,^^147^ but a large GWAS of 4571 patients failed to replicate the former and reported opposite-direction effects for the latter.^148^ However, variants in *ARRB1*, which encodes *β*-arrestin-1, are associated with greater GLP-1RA effects, suggesting that receptor internalization dynamics may contribute to response variability. Given the links between *β*-arrestin signaling and nausea,^149^ and observations that small-molecule GLP-1RAs reduce *β*-arrestin recruitment,^150^ downstream signaling variation may influence both efficacy and tolerability.

Although no pharmacogenetic markers currently guide incretin therapy selection, genomic analyses have yielded valuable mechanistic insights.^151^^,^^152^ Mendelian randomization analyses suggest that GLP-1 receptor activation exerts cardioprotective effects beyond glycemic improvement,^153^ and that GIPR variants are associated with favorable cardiometabolic and liver outcomes.^154^^,^^155^ Overall, individual variants explain only a minor fraction of incretin response variability, but polygenic approaches may improve predictive power in the future.

### D Cardiovascular disease

Cardiovascular therapeutics have been a major focus of pharmacogenomic research over the past 15 years.^156^ Several drug-gene pairs now have strong mechanistic evidence, clinical validation, and formal implementation guidelines. The most mature examples involve clopidogrel, warfarin, and statins, each with reproducible genotype–phenotype associations and meaningful impacts on safety or efficacy.

#### 1 Clopidogrel and CYP2C19

The most robustly validated cardiovascular pharmacogenomic application is CYP2C19-guided antiplatelet therapy. Clopidogrel requires CYP2C19-mediated activation. Loss-of-function alleles such as *CYP2C19∗2* and *CYP2C19∗3* inhibit the formation of the active metabolite, leading to diminished platelet inhibition and an increased incidence of major adverse cardiovascular events in acute coronary syndrome and percutaneous coronary intervention.^157^^,^^158^ IMs and PMs consistently show elevated on-treatment platelet reactivity and increased risks of stent thrombosis and recurrent ischemia despite standard dosing. Consistent with this evidence, CPIC recommends avoiding clopidogrel in CYP2C19 IMs and PMs with acute coronary syndrome or undergoing percutaneous coronary intervention, and instead using prasugrel or ticagrelor when appropriate.^159^ NMs, rapid metabolizers, and UMs may continue standard therapy.

#### 2 Warfarin and VKORC1/CYP2C9/CYP4F2

Warfarin is another extensively characterized drug of pharmacogenomic interest. Variation in VKORC1, CYP2C9, and CYP4F2 alters therapeutic dose requirements. VKORC1 −1639G>A modifies transcriptional regulation of the target enzyme. Reduced-function CYP2C9 alleles *(∗2, ∗3, ∗5, ∗6, ∗8,* and *∗11*) impair *S*-warfarin clearance and increase bleeding risk during initiation.^160^ *CYP4F2∗3* increases vitamin K recycling, modestly increasing dose requirements.^161^ Together, these variants account for 30%–40% of dose variability in European and Asian populations, with somewhat smaller but still meaningful contributions in individuals of African ancestry.^162^

Multiple randomized and observational studies show that genotype-guided dosing improves the accuracy of initial dose selection and reduces early supratherapeutic anticoagulation.^163^ CPIC guidelines incorporate genetic and clinical variables into ancestry-specific dosing algorithms and represent one of the most advanced examples of pharmacogenomic implementation in clinical practice.^160^ Overall, the shift in anticoagulant therapy from warfarin to protein inhibitors makes the pharmacogenomic component of anticoagulant therapy less clinically important.

#### 3 Statins and SLCO1B1

Statin therapy is another high-priority pharmacogenomic domain, driven primarily by variation in *SLCO1B1*, which encodes OATP1B1, with additional roles for ABCG2 and CYP2C9. The *SLCO1B1* c.521T>C variant reduces hepatic statin uptake, increasing systemic exposure and substantially elevating risk of statin-associated muscle symptoms, particularly with simvastatin.^164^ *ABCG2* c.421C>A and *CYP2C9* reduced-function alleles influence exposure to rosuvastatin and fluvastatin, respectively.^165^ CPIC guidelines recommend avoiding high-dose simvastatin or using alternative statins in individuals with high-risk *SLCO1B1* genotypes while emphasizing that genotype information should optimize therapy rather than deter statin use.^166^

#### 4 β-blockers

Compared with clopidogrel, warfarin, and statins, the pharmacogenomic evidence for *β*-blockers remains moderate and insufficient for guideline-driven implementation. CYP2D6 PMs show increased metoprolol exposure and a higher incidence of bradycardia or hypotension, while UMs may experience a reduced therapeutic response.^167^ A 2020 meta-analysis of 15 studies (*n* = 1146) reported that PMs had only a modestly greater reduction in heart rate, approximately 3 bpm (*P* = .017), an effect generally considered too small to be clinically actionable.^168^ Indeed, the recent CPIC guideline discusses these findings and concludes that CYP2D6 polymorphisms exert relatively modest clinical effects on metoprolol and other *β*-blockers.^169^

Pharmacodynamic variants in ADRB1, ADRB2, and GRK5 have also been suggested to influence the response to *β*-blockers. ADRB1 p.R389= carriers often demonstrate greater sensitivity to *β*-blockers, while GRK5 Leu41 may confer “genetic *β*-blockade,” particularly in individuals of African ancestry.^170^ Although consistent across many cohort studies, variation in clinical endpoints and the absence of large, prospective, genotype-guided trials limit translation into recommendations, as also supported by the CPIC guideline.^169^

#### 5 Other possible biomarkers

A broader set of cardiovascular pharmacogenomic associations is under investigation, including variants that affect renin-angiotensin-aldosterone system inhibitors, direct oral anticoagulants, antiarrhythmics, and predisposition to drug-induced arrhythmias. While mechanistic and association studies highlight potential relevance, current evidence does not support clinical implementation, and no consensus guidelines exist.

Overall, cardiovascular pharmacogenomics has progressed from mechanistic investigation to clinically actionable guidance for several high-impact drug classes. The strongest evidence supports genotype-guided prescribing for clopidogrel, warfarin, and statins, whereas biomarkers are of less value for *β*-blocker therapy.

### E Oncology

Implementation of pharmacogenomic biomarkers is widespread in oncology. Most factors pertain to somatic variants that inform targeted therapies and are outside the scope of this review. We thus refer the interested reader to recent reports on this topic.^171^, ^172^, ^173^ In addition to variations in the cancer genome, multiple germline variants have emerged as robust biomarkers that associate with altered drug disposition. These include *DPYD* for fluoropyrimidines, *UGT1A1* for irinotecan, *TPMT* and *NUDT15* for thiopurine drugs, and *CYP2D6* for tamoxifen. Besides their individual use to inform drug selection and dosing of chemotherapeutics, there are also increasing efforts to leverage both somatic and germline variants within integrated workflows to guide pharmacotherapy in precision oncology.^174^

#### 1 DPYD polymorphisms

*DPYD,* which encodes dihydropyrimidine dehydrogenase, is critical for the catabolism of fluoropyrimidines. Reduced dihydropyrimidine dehydrogenase activity leads to accumulation of active fluoropyrimidines, increasing the risk of myelosuppression, mucositis, and gastrointestinal toxicity. Well characterized reduced-function variants include the splice-donor variant allele *DPYD∗2A* (rs3918290), the missense variants DPYD p.I560S (rs55886062) and p.D949V (rs67376798), and the intronic splice variant rs75017182 with its associated haplotype HapB3.^175^, ^176^, ^177^, ^178^ Multiple clinical guidelines now recommend *DPYD* genotyping before starting fluoropyrimidine therapy, and genetically guided fluoropyrimidine therapy is increasingly implemented in Europe^179^ and the United States.^180^^,^^181^ Furthermore, a multitude of rare variants can affect fluoropyrimidine sensitivity.^182^, ^183^, ^184^ While testing for such rare single variants at the population level is currently not advised, extending from candidate genotyping to sequencing-based approaches does offer benefits for identifying at-risk variants at the individual level to explain and possibly predict fluoropyrimidine-related severe toxicity.^185^^,^^186^

#### 2 Variability in UGT1A1

Another established marker is the *UGT1A1* variant, which guides irinotecan treatment. UGT1A1 mediates the glucuronidation of SN-38, the active metabolite of irinotecan, and patients in which SN-38 clearance is impaired are at elevated risk of severe neutropenia and diarrhea. The clinically most important variant concerns the TATA box of the *UGT1A1* promoter. While the normal allele has 6 TA repeats, insertion alleles such as *UGT1A1∗28* (TA_7_) and *UGT1A1∗37* (TA_8_) result in reduced UGT1A1 expression and activity. Inversely, a shortening of the TATA box, as in *UGT1A1∗36* (TA_5_), results in increased activity. *UGT1A1∗28* is very common globally (minor allele frequency [MAF] of 30%–45%), except in East Asian populations, where its frequency is considerably lower (MAF = 12%).^187^ In contrast, *UGT1A1∗36* and *UGT1A1∗37* are limited to African populations (MAF = 7% and 5%, respectively; <1% in other populations), and the reduced-function missense variant *UGT1A1∗6* (p.G71R, rs4148323) is common in East Asia (MAF = 16%) and Europe (MAF = 4%). The available clinical guideline from the DPWG suggests a 70% starting dose of irinotecan for patients homozygous or compound heterozygous for the reduced-activity alleles *UGT1A1∗6*, *∗28,* or *∗37,* and this testing is considered essential for drug safety.^188^ However, neither the EMA nor the FDA has provided firm guidance that would mandate pre-emptive testing as of yet.^189^

#### 3 Thiopurines

Thiopurines, used in acute lymphoblastic leukemia and other nonmalignant immunological indications, such as inflammatory bowel disease and rheumatoid arthritis, can be subject to dose-limiting myelotoxicity, which can be life-threatening. TPMT and nudix hydrolase 15 (NUDT15) partake in the inactivation of thiopurine metabolites, and variations in the genes encoding these enzymes are the most important determinants of interindividual variation in thiopurine metabolism and toxicity. The *TPMT* alleles *TPMT∗2*, *∗3A,* and *∗3C* are the main actionable variants in individuals of European and African ancestry, whereas the *NUDT15* variants *NUDT15∗2*, *∗3*, and *∗9* play a dominant role in Asian populations.^190^^,^^191^ Overall, it is estimated that TPMT and NUDT15 deficiency account for approximately 35% and 15% of all thiopurine-related adverse events, respectively.^192^^,^^193^ Based on the available evidence, there is a strong recommendation to reduce thiopurine doses in carriers of reduced-activity alleles. In heterozygotes, a 30%–70% dose reduction is typically advised, while doses ≤10% of the standard and an increase in the time window to reach steady-state from 2 weeks to 4–6 weeks before dose adjustments are recommended in poor TPMT or NUDT15 metabolizers^194^; these guidelines are, however, not prescriptive at present.

#### 4 Tamoxifen

Tamoxifen is the key adjuvant therapy for estrogen receptor-positive breast cancer, reducing recurrence and improving survival. Its effectiveness depends on hepatic conversion to active metabolites, especially endoxifen, which is mainly catalyzed by CYP2D6.^195^ However, the effects of endoxifen levels on clinical outcomes remain controversial, with some studies identifying greater benefits in patients with plasma levels >9–16 nM,^196^, ^197^, ^198^ whereas others do not find significant associations between endoxifen levels and *CYP2D6* genotype and relapse or survival.^199^^,^^200^

*CYP2D6* is highly polymorphic, with over 180 known alleles, and genotype strongly correlates with plasma endoxifen levels.^201^ A recent cross-ancestry GWAS of German, Chinese, and Lebanese patients found that only the *CYP2D6* locus was associated with endoxifen metabolic ratios (*P* = 2 × 10^-65^), with no other loci reaching genome-wide significance.^56^ Pharmacokinetic differences linked to CYP2D6 can influence breast cancer recurrence and mortality during tamoxifen therapy, supported by retrospective studies^202^^,^^203^ and meta-analyses.^204^ Current guidelines recommend increasing tamoxifen from 20 to 40 mg/d for PMs and IMs,^205^ though PMs may still be underdosed.^206^ The recent TAMENDOX trial tested 3 mg daily endoxifen supplementation and showed that 12 of 13 PMs (92.3%) reached therapeutic endoxifen levels (>32 nM), compared with 0 of 9 without supplementation.^207^ Overall, evidence supports *CYP2D6* genotype as a major determinant of tamoxifen metabolism and clinical outcome.

#### 5 Immunotherapy

One of the newer areas of research is how germline variation influences outcomes and toxicity in immunotherapy. There is increasing interest in how inherited variants in immune regulation and inflammatory responses may affect the efficacy of immune checkpoint inhibitors or risk of immune-related adverse events. These include polymorphisms in *PDCD1* (PD-1), *CD274* (PD-L1), *CTLA4,* and related genes, as well as variations in the major histocompatibility complex.^208^, ^209^, ^210^ Although these are still exploratory, there is increasing evidence that germline markers in oncology may also play roles beyond influencing drug pharmacokinetics.

### F Infectious diseases

Pharmacogenomic biomarkers can be useful to guide the treatment with various antiretrovirals, antimalarials, and antifungal medications. Arguably, the most established pharmacogenomic example is the association of *HLA-B∗57:01* with hypersensitivity to the nucleoside reverse transcriptase inhibitor abacavir, which is used to treat human immunodeficiency virus infection. Mechanistically, the drug molecule binds to the peptide-binding groove of the HLA variant, resulting in changes to the repertoire of presented peptides, which, in turn, activate T cells and cause autoimmunity.^211^ Strikingly, the risk of abacavir hypersensitivity is drastically increased in *HLA-B∗57:01* carriers, with typical ORs of 50–1000, whereas noncarriers are completely protected (negative predictive values of 100%).^212^ Due to the substantial socioeconomic costs associated with abacavir adverse events, *HLA-B∗57:01* testing has been found to be cost-effective in most populations, except for East Asian populations (China, Japan, and South Korea), where the risk allele is very rare.^213^ Consequently, pre-emptive screening for *HLA-B∗57:01* prior to initiating abacavir is mandated by the FDA and EMA for abacavir-naïve patients, but not by the Japanese regulatory agency, the Japanese Pharmaceuticals and Medical Devices Agency.

#### 1 Reverse transcriptase inhibitors

Besides abacavir, pharmacogenomic associations have also been reported for other nucleotide and nonnucleotide reverse transcriptase inhibitors, such as tenofovir and efavirenz. For tenofovir, variations in various transporter genes, such as *ABCC2*, *ABCC4*, *SLC22A6,* and *SLC28A2,* have been reported to impact pharmacokinetics and human immunodeficiency virus nonsuppression, with combined effect sizes up to 48^214^^,^^215^; however, these reports lack replication and prospective validation. Efavirenz is subject to high interindividual variability in plasma concentrations, which are associated with therapeutic efficacy and neurological adverse events. The primary determinant of this variability is genetic variation in *CYP2B6*, which encodes the hepatic enzyme responsible for efavirenz clearance. In particular, the *CYP2B6*∗*6* and *CYP2B6∗18* alleles have been shown to reduce metabolism, leading to higher efavirenz plasma levels,^216^ which increase protection against viral breakthrough^217^ but also increase the risk of neurological toxicity and drug discontinuation.^218^^,^^219^ Importantly, the spectrum of genetic variability in *CYP2B6* differs drastically across populations. Frequencies of *CYP2B6*∗*6* and *CYP2B6∗18* are highest in African populations, with MAFs of up to 45% and 12%, respectively,^220^^,^^221^ with important implications for public health. A striking example is the rollout of efavirenz in Zimbabwe in 2015, following recommendations and guidelines from the World Health Organization.^222^^,^^223^ Upon switching to efavirenz, many Zimbabweans experienced unanticipated neurological adverse events, consistent with efavirenz overdose. The reason for this public health disaster was that the frequency of *CYP2B6∗6* was exceedingly high in Zimbabwe, with a MAF of 49% and 20% of individuals being homozygous.^224^ As the pivotal efavirenz trials were conducted outside Africa,^225^, ^226^, ^227^ these findings serve as an important warning that consideration of ethnogeographic genetic context is critical for selecting effective and safe therapies.

#### 2 Treatment of malaria

In addition to the impact of antiretroviral therapy, pharmacogenetic biomarkers have been established for malaria treatment. Artemisinin-based combination therapy is the recommended first-line treatment for uncomplicated *Plasmodium falciparum* malaria, combining a fast-acting artemisinin derivative, such as artemether or artesunate, with a longer-acting drug, such as lumefantrine, piperaquine, or amodiaquine. Only a few small studies (<80 participants) have reported pharmacogenetic associations with artemisinin derivative efficacy, including reduced-function alleles in *CYP2B6*^228^ and the possibly increased-function allele *CYP2A6∗46* (previously known as *CYP2A6∗1B*).^229^ In contrast, there is increasing evidence for an association between the efficacy of long-acting amodiaquine and variations in *CYP2C8*, which encodes the enzyme that catalyzes *N-*deethylation to the main active metabolite, desethyl-amodiaquine. *CYP2C8∗2* is the most common reduced-function allele in Africa, while *CYP2C8∗3* is the most common in European populations.^230^ While these alleles have not been shown to affect amodiaquine efficacy, they are associated with a significant increase in the frequency of mild adverse events.^231^^,^^232^ This can reduce compliance when used as seasonal malaria chemoprevention regimens and thus indirectly impact malaria treatment success and the emergence of drug resistance.^233^

For *Plasmodium vivax*, primaquine is the only drug that eliminates the dormant hypnozoite liver-stage forms of the parasite. Primaquine is a prodrug that requires CYP2D6-mediated bioactivation to form its hydroxylated active metabolites. The defective *CYP2D6* alleles, such as *CYP2D6∗4*, *∗5,* and *∗10*, have been robustly linked to treatment failure and *P vivax* recurrence across different populations, with ORs ranging from 2.1 to 6.5.^234^, ^235^, ^236^, ^237^ Primaquine safety is strongly linked to G6PD deficiency, which is caused by deleterious variants in the X-linked *G6PD* gene. G6PD deficiency affects ∼5% of the global population, with the highest frequencies in Africa (up to 12.4% in males), moderate in South Asia, and the lowest in Europeans (<1%).^238^ The condition spans multiple severity categories. Individuals with <30% enzyme activity are at high risk of primaquine-induced hemolytic anemia, while those with 30%–70% may require dose modification and monitoring.^239^ This challenge is significant because G6PD deficiency is concentrated in malaria-endemic regions.^240^ Therefore, *G6PD* genotyping or enzyme activity testing is recommended before primaquine use.^241^ Notably, 3-week regimens may offer a safer, effective alternative for G6PD-deficient patients.^242^

#### 3 Fungal treatment

Voriconazole is a triazole antifungal mainly used for invasive *Aspergillus* and *Candida* infections. It exhibits striking interindividual variability in plasma concentrations, therapeutic efficacy, and toxicity, which are at least in part related to variations in *CYP2C19*.^243^, ^244^, ^245^ The *CYP2C19* loss-of-function allele *CYP2C19∗2* is very common in Asian populations (MAF = 30%–35%), whereas the increased-function allele *CYP2C19∗17* is less frequent than in Europeans or Africans (MAF = 1.3%–13.6% vs 22%–23% in Africans and Europeans).^246^ In a recent study from Japan, voriconazole doses were adjusted based on *CYP2C19* genotype, resulting in a significantly lower incidence of adverse events (*P* = .003) without negatively affecting response rates.^247^ Combined, these results indicate that voriconazole is a promising candidate for pharmacogenetically guided prescribing, particularly in Asia. However, nongenetic factors, such as inflammation, can lead to marked phenotypic conversion and should therefore be considered when optimizing individualized treatment.^248^

### G Pharmacogenomic determinants of analgesic response

#### 1 CYP2D6-mediated formation of opioids

Several widely used analgesics, most prominently codeine and tramadol, and, to a lesser degree, hydrocodone, require CYP2D6-mediated *O-*demethylation to generate their pharmacologically active metabolites. As a result, the CYP2D6 genotype exerts a direct, quantifiable influence on opioid exposure-response relationships. Individuals with PM status exhibit markedly reduced formation of morphine or *O-*desmethyltramadol, whereas UMs accumulate supratherapeutic metabolite levels with standard dosing.^249^ A recent study by Ashraf et al^250^ elegantly demonstrates the relationship between the *CYP2D6* genotype and the codeine metabolism phenotype in a Finnish population, which notably exhibits a high (6%) frequency of *CYP2D6* UM alleles.

In a cohort exceeding 31,000 adults prescribed CYP2D6-dependent opioids, individuals with reduced functional CYP2D6 activity, whether genetically determined or induced via phenoconversion through coadministration of strong CYP2D6 inhibitors, experienced significantly higher rates of pain-related emergency department visits.^251^^,^^252^ This reinforces the concept that an effective CYP2D6 phenotype, rather than genotype alone, governs opioid responsiveness.

#### 2 Genetic Influences on pain sensitivity and opioid requirement

Analgesic response is influenced by genetic determinants of baseline nociceptive processing. One of the most thoroughly studied receptor-level variants, the *μ*-opioid receptor (OPRM19 p.A118G [rs1799971]), linked to reduced receptor expression and lower agonist binding efficacy, has consistently been associated with altered opioid potency requirements. A recent meta-analysis of obstetric analgesia confirmed higher opioid consumption among carriers of the G allele relative to AA homozygotes.^253^ The evidence is strong enough to suggest it influences opioid response, but not strong enough to justify changes in prescribing protocols solely based on genotype.

#### 3 Pharmacogenomic determinants of nonopioid analgesic response

*CYP2C9* polymorphisms have a well defined impact on the disposition of nonsteroidal anti-inflammatory drugs (NSAIDs) and on gastrointestinal toxicity. Reduced-function alleles (∗2 and ∗3) significantly diminish CYP2C9 catalytic activity, resulting in decreased clearance and proportionally increased systemic NSAID exposure.^254^ This exposure elevation enhances cyclooxygenase-1 inhibition and thereby intensifies suppression of gastroprotective prostaglandins, increasing susceptibility to erosion, ulceration, and upper gastrointestinal bleeding. Recent implementation-focused analyses indicate that *CYP2C9* remains the most robust pharmacogenetic determinant of NSAID toxicity risk across perioperative and chronic pain settings, with ∗3/∗3 carriers exhibiting the highest predicted exposure and toxicity burden.^255^ Ethnogeographic studies similarly demonstrate substantial variability in *CYP2C9* decreased-function allele frequencies across global populations, emphasizing the need for population-sensitive risk stratification in NSAID prescribing.^132^ Collectively, current evidence supports *CYP2C9*-guided dose adjustment or alternative analgesic selection in patients at elevated risk of NSAID-associated gastrointestinal complications.

## VIII Emerging in silico methods for pharmacogenomic research

Pharmacogenomic investigations of interindividual variability in drug response mostly rely on epidemiological associations between genetic variations and pharmacological phenotypes. To provide mechanistic support and test emerging hypotheses, experimental systems are critical complements. Developments over the last decade have been accelerated by converging trends. First, novel pharmacogenomic variant effect predictors^256^ and emerging experimental methods, such as deep mutational scanning,^257^ enable systematic annotation of the functional impact of genetic variants in ADME genes and drug targets. Second, the establishment and increasing adaptation of physiologically relevant 3-dimensional (3D) human tissue models that closely resemble their in vivo counterparts at the molecular and functional levels enable the emulation of human drug ADME, response, and toxicity with unprecedented accuracy. Third, advances in machine learning and mechanistic modeling strategies enable the use of these rich experimental readouts to build predictive models of pharmacokinetics, pharmacodynamics, and toxicity.

Interindividual differences in drug response arise largely from genetic variation in ADME genes and drug targets. More than 70,000 coding variants have been described in ADME genes,^258^, ^259^, ^260^ and more than 30,000 in drug targets.^261^ Furthermore, ADME genes harbor almost 5000 CNVs^82^ and nearly 1200 structural variants that can alter pharmacogene expression by affecting gene regulatory elements.^83^ Importantly, only a minute fraction of these variants has been functionally characterized, leaving the consequences of the majority of these variants uncertain. Experimental follow-up has been slow and candidate-driven, resulting in a biased understanding centered on established pharmacogenes and an overrepresentation of variants common in European populations.

### A In silico prediction of functional genetic variability

Computational variant effect predictors were designed to classify mutations as either pathogenic or benign, typically using evolutionary constraints as their central metric. Typical algorithms in this category include SIFT,^262^ PolyPhen-2,^263^ and CADD.^264^ This strategy, however, is poorly suited for pharmacogenomics, because only a small subset of pharmacogenes are linked to inherited diseases, meaning that “pathogenicity” is not an appropriate proxy for functional impact on drug response.^265^ Foundation protein language models, such as ESM,^266^ ProGPT2,^267^ ProGen,^268^ and AlphaMissense,^269^ improve predictions by learning mutational tolerance directly from millions of natural protein sequences. While a detailed evaluation of their performance on pharmacogenes has not been presented, their increasing focus on protein structure and stability suggests that they are likely to outperform conventional models that rely primarily on sequence conservation.^270^

From a computational perspective, these shortcomings were addressed through the development of effect predictors specifically for pharmacogenes that use structural and functional genomic data as key parameters for variant classification. Some of these are tailored to individual genes, such as the DPYD-Varifier for variants in *DPYD*^271^ or the CYP2D6-specific algorithm Hubble.2D6.^272^ Other tools have been developed to predict functional effects across the entire pharmacogenome. These include the ADME-optimized prediction framework (APF), which was trained on a curated dataset of 337 experimentally tested variants spanning 44 pharmacogenes.^273^ More recently, the framework has been enhanced through the incorporation of AlphaFold-based structural impact estimates.^274^ The resulting APF2 generates quantitative variant effect scores that strongly correlate with empirical measurements (*R*^*2*^ = 0.91; *P* = .003), leading to superior predictive accuracy, especially for clinically actionable variants covered by pharmacogenetic guidelines. In addition, the APF algorithm accurately predicted the clinical consequences of *DPYD* and *TPMT* variants (APF accuracy of 91.4%, compared with 95.3% in vitro), despite not being used in model development.^190^ Because the correlation between variant effect predictors and functional assays was found to be a good proxy of their performance in clinical applications,^275^ these results suggest that structure- rather than conservation-based effect prediction can reliably estimate the functional consequences of newly identified variants of unknown significance.

### B Deep mutational scanning

The advent of deep mutational scanning has enabled the systematic experimental characterization of hundreds to tens of thousands of pharmacogenetic variants in parallel using recombinant expression systems.^276^ These powerful approaches have already systematically profiled variant effects on activity or abundance in pharmacokinetic genes, such as *CYP2C9*,^277^ *CYP2C19*,^278^ *NUDT15*,^279^ *SLC22A1*,^280^ and *TPMT*,^281^ as well as in multiple drug targets, including *VKORC1*,^282^ *ADRB2*,^283^ and *MC4R*.^284^ The increasing adaptation of these assays is supported by an array of publicly available computational tools for data processing and variant effect scoring.^285^ In a recent study that involved the massive parallel evaluation of more than 13,000 variants in the insulin receptor, the authors identified variants that affect receptor surface expression, insulin binding, and downstream signaling.^286^ These results reveal insights into insulin binding dynamics and pinpoint the molecular mechanisms underlying variant pathogenicity. On an even larger scale, evaluation of the effects of 500,000 variants on the abundance of more than 500 human protein domains revealed how mutational effects can impact protein stability and what these changes mean for protein fitness.^287^ Together, these studies show how an exhaustive characterization of the protein variant landscape can yield new insights into gene-function relationships to guide the development of novel therapeutics, demonstrating the power of deep mutational scanning for functional genomics of pharmacokinetic and pharmacodynamic genes.

Coupling deep mutational scanning scores with population-scale sequencing and outcome data, eg, via the UK or Estonian Biobanks, enables association testing between experimentally defined loss-of-function or gain-of-function classes and real-world phenotypes, such as dose requirements, adverse drug reactions, or treatment failure. Pharmacogenomic in silico predictors, such as APF, then fill in missing data for variants that have not yet been experimentally assessed, enabling comprehensive in silico saturation mutagenesis of the entire pharmacogenome. This approach aims to transform pharmacogenomics from the selective evaluation of candidate variants to a systematic discipline that provides evidence-based decision support for every patient with available sequencing data.

## IX Organotypic human tissue models as emerging enablers

Besides advances in computational tools and experimental methods using recombinant cell systems, there have been important developments in physiologically relevant 3D human tissue models.^288^, ^289^, ^290^ These systems include stem cell-derived organoids, spheroids typically derived from fully differentiated mature cells, and precision-cut slide cultures. Organoids derived from induced pluripotent stem cells (iPSCs) or adult stem cells have been established for a wide range of tissues and organs, including, but not limited to, the intestine,^291^ liver,^292^ kidney,^293^ and brain.^294^ Through a series of differentiation steps that can take from a few days to months, stem cells are expanded and differentiated in a stepwise process to give rise to specific cell types. These increasingly complex protocols can now generate not only individual cell types but also recapitulate at least part of the cellular complexity of the modeled tissue. For instance, intestinal organoids can generate not only enterocytes but also goblet, Paneth, and enteroendocrine cells.^295^ Moreover, recent protocols allow balancing differentiation and proliferation of human intestinal organoids in a single culture condition, without the need for sophisticated gradients.^296^

### A Organoids

Organoid cultures are well suited for highly proliferative tissues, such as the intestine; however, for slowly proliferative tissues, complete differentiation has not yet been achieved, and phenotypes remain fetal. Too short culture and differentiation times have been discussed as a likely reason for this lack of maturity. For instance, hepatocytes can live for decades,^297^ and liver development continues postnatally for many years, giving rise to “final” mature hepatocytes.^298^

Despite the overall lack of functional maturity, organoids have enabled unique approaches to pharmacogenomic investigations. For instance, by establishing hepatocyte-like cells from iPSCs derived from patients who experienced hepatotoxicity during pazopanib treatment, it was possible to identify the molecular mechanisms of pazopanib toxicity.^299^ Due to the highly invasive nature of liver biopsies, this study was only possible because iPSCs could be generated from B lymphocytes by episomal reprogramming (ie, only requiring a conventional blood draw), thus demonstrating how stem cells can help link clinical phenotypes to patient-specific molecular alterations. Another notable example is the confirmation of a polygenic risk score for sensitivity to drug-induced liver injury (DILI) in liver organoids from 5 donors.^300^ The results confirmed the genetic architecture of DILI, identified through the integration of multiple large-scale GWAS, thereby providing a scalable experimental toolkit for exploring complex genetic associations with DILI sensitivity across multiple drugs. In summary, organoids are well suited for (1) tissues with a natural high turnover rate, (2) studies of tissue and organ development, (3) studies in which donor material is very difficult to assess, eg, due to the highly invasive nature of tissue sampling and the lack of donor tissue, and (4) applications based on genetic manipulation. In contrast, we consider their utility limited to studies of chronic disease, applications that require mature tissue function, and complex models that require integration of complex immune cell repertoires.

### B Spheroids

An alternative to organoids is spheroid culture. In this paradigm, fully differentiated, mature cells isolated from biopsies, resections, or organ donations are cultured in 3D aggregates. Spheroid culture is extensively used for a multitude of tissues, including liver,^301^ adipose,^302^ and pancreatic islets.^303^ Spheroids can support the functional and phenotypic maintenance of cultured cells at near-physiological levels for multiple weeks to months. Furthermore, they offer the possibility of integrating complex patient-derived immune cell compositions, including macrophages, T cells, B cells, and natural killer cells, which cannot be generated from stem cells.^304^ From a pharmacogenomic perspective, spheroid culture is particularly relevant to the liver, where hepatocytes maintain their full complement of drug-metabolizing enzymes and transporters,^305^^,^^306^ rendering them useful for investigating the effects of genetic variation on drug disposition. For instance, comparison of the metabolic fluxes of the CYP2D6 substrate dextromethorphan in liver spheroids from NMs and PMs of CYP2D6 demonstrated a shift in metabolite abundances away from dextrorphan toward 3-methoxymorphinan.^307^ While we are not aware of similar investigations of genetic variations in drug transporters, the compatibility of spheroids with hepatic uptake and secretion assays would render such studies technically feasible.^308^

In contrast to the aforementioned tissues, spheroid culture is less suited to epithelial tissues such as the intestine, skin, or blood-brain barrier,^309^ tissues where mature cells cannot be reliably obtained, such as the cerebellum, or tissues that require the formation of complex anatomical structures to recapitulate function, such as the kidney. Furthermore, although knockdown and overexpression are highly efficient, it is important to note that genetic manipulation using CRISPR/Cas9 or similar base editors is not feasible due to the lack of clonal expansion steps.

### C Coping with bias factors

A recurring challenge in pharmacogenomics is distinguishing genetic effects from environmental confounders. 3D models enable experimental designs that parallel human cohorts while maintaining experimental control. In the future, we envision that establishing donor panels and biobanks of culturable cells with linked genotypes will enable experimental testing of epidemiological associations and yield important mechanistic insights. These repositories should contain dozens to hundreds of donors and ensure representation across ancestries to capture allele-frequency differences and linkage disequilibrium structure. Deep phenotyping that combines functional endpoints, such as intrinsic clearance, transepithelial electrical resistance, and transporter activity, with multiomics and morphological profiling based on high-content imaging and cell painting promises to capture latent phenotypes linked to genetic variation. Importantly, these high-dimensional, longitudinal data streams can be integrated with artificial intelligence (AI) to improve pharmacokinetic modeling and early risk assessment. For instance, deep learning of physicochemical compound features, pharmacokinetic parameters, and embeddings of omics and morphology data has drastically improved the accuracy of DILI prediction.^310^, ^311^, ^312^ Furthermore, deep learning frameworks based on longitudinal imaging data that involve representative learning, where a convolutional neural network extracts feature embeddings and metric learning that transforms the feature space to cluster similar images across the time series, have been successful in predicting the mechanisms of action of new compounds based on subcellular phenotypes.^313^ Thus, the combination of organotypic 3D human culture systems, deep phenotyping, and generative AI offers exciting new opportunities for predicting and mechanistically understanding pharmacogenomic associations. By leveraging increasingly large multimodal datasets, these frameworks can serve as digital twins, ie, virtual replicas of a tissue, organs, or entire organisms of interest, that can guide mechanistic modeling approaches and, eventually, precision medicine in practice.^314^^,^^315^

## X Outlook

The field of pharmacogenomics is rapidly advancing, both in terms of methodologies for identifying and characterizing genetic variants and in the development of AI-driven tools that enhance the functional interpretation of genetic data and its clinical translation. A search of ClinicalTrials.gov reveals that more than 300 pharmacogenomics-related clinical trials are currently in progress. Historical trends from ClinicalTrials.gov show a steady increase in PGx-related studies since the early 2000s, particularly in oncology and psychiatry.^316^ Many of these clinical trials focus on *CYP2D6* in psychiatry and pain management, *CYP2C9* in hematology, *CYP2C19* in cardiology and psychiatry, *CYP3A5* in transplantation, and *UGT1A1* in oncology. Based on experience from recent large pharmacogenomic trials, it is evident that these studies must anticipate and mitigate several methodological pitfalls, including (1) too few patients carrying actionable variants that are usually rare, (2) uncontrolled placebo effects due to the absence of consistent blinding and labeling, (3) nonoptimal selection of gene-drug pairs, (4) insufficient evaluation of liver and kidney pathology, (5) low patient adherence, and (6) unmonitored drug–drug interactions.

Relevant regulatory frameworks support better study design and data interpretation. For example, the EMA Guideline on Good Pharmacogenomic Practice (EMA/CHMP/718998/2016) provides methodological standards,^317^ and the Pharmacogenomic Data Submissions Draft Guidance for Industry offers updated direction on when pharmacogenomic findings must be included in Investigational New Drug, New Drug Application, or Biologics License Application submissions, and on how such data should be reported.^86^ It is anticipated that introducing pharmacogenomic analyses early in the drug development process will decrease the number of drugs that reach the market for which such analyses later prove essential to predict efficacy and adverse reactions.

**Fig. 4:**
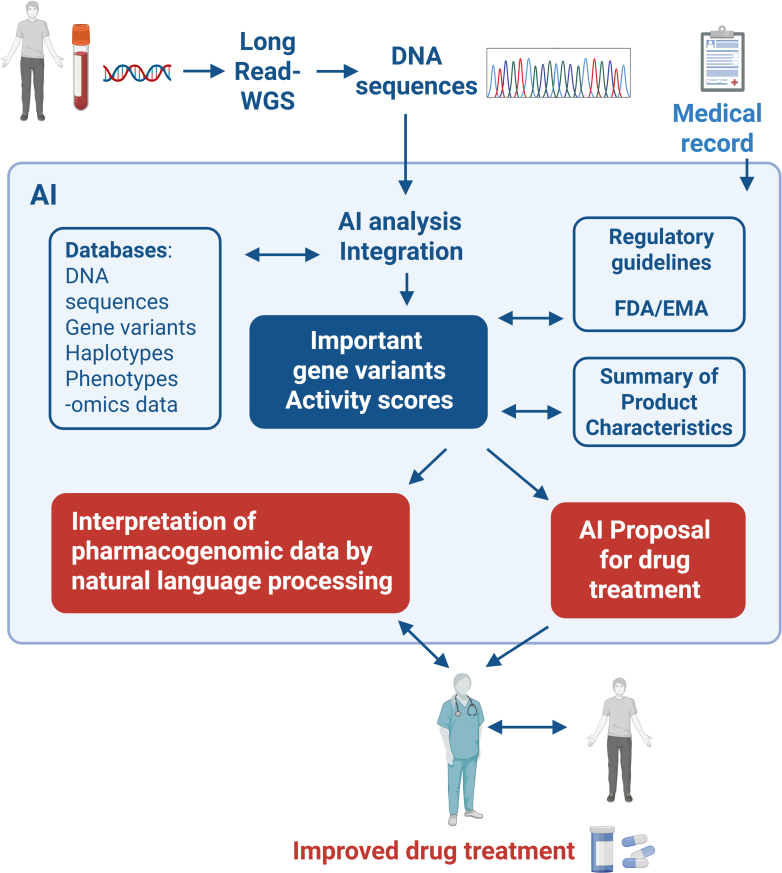
The increasing use of AI in pharmacogenomics. AI supports pharmacogenomic implementation at multiple levels by enabling rapid whole-genome sequencing (WGS), interpreting complex genomic data, and extracting key insights from scientific literature, databases, regulatory product labels, pharmacogenomic guidelines, and clinical records. These insights are translated into actionable recommendations that support clinical decision-making. AI-driven natural language processing further helps clinicians adapt this information to the individual patient, resulting in improved drug prescribing and better treatment outcomes.

A major challenge is the integration of pharmacogenomics into routine clinical practice. Clinicians require sufficient education in the field, pharmacogenomic data must be seamlessly incorporated into electronic health records, and large, well powered studies focusing on specific gene-drug pairs are essential. In this context, AI can support implementation at multiple levels (Fig. 4). It enables rapid whole-genome analysis, helping prioritize haplotypes and clinically relevant gene-variant-drug interactions. AI-driven natural language processing further assists clinicians by interpreting complex genomic data, extracting key insights from research and medical records, and translating them into clear, actionable guidance for patient care. AI can also integrate pharmacogenomic data with other omics, such as transcriptomics, proteomics, and metabolomics, to build a more comprehensive understanding of drug response mechanisms. Furthermore, it helps detect and mitigate biases in genomic datasets, leading to more equitable and effective treatment outcomes.

From a hardware perspective, rapid advances are underway, and it is likely that whole-genome sequencing will soon be routinely available in clinical settings, providing comprehensive genomic data at substantially lower cost. In conclusion, pharmacogenomics offers a valuable tool for optimizing the prescription of approximately 60–100 drugs, but it must be applied carefully, with attention to minimizing bias and ensuring equitable interpretation. Current cost-benefit analyses remain inconclusive and require further evaluation through robust, well designed clinical studies.

Overall, we are happy that the field of pharmacogenomics, over its quarter-century of development, has advanced in parallel with technological progress to become a mature and powerful tool for improving drug therapy. Today, however, one of the greatest challenges is translating pharmacogenomic knowledge and methodology into routine clinical practice across all areas of medicine, both in specialist settings and in general healthcare. Thus, we believe that guidance from regulatory authorities must become clearer and more harmonized across agencies, and that pharmacogenomic drug labels should be continuously updated to reflect emerging evidence. Furthermore, education and training in pharmacogenomics need to be strengthened throughout the healthcare sector to ensure that clinicians at all levels are equipped to apply this knowledge in patient care. The cost-benefit of pharmacogenomic advice should be continuously evaluated. Only through coordinated regulatory clarification, transparent communication, and strengthened professional education can the full potential of pharmacogenomics be realized for the benefit of public health.

## Conflict of interest

Volker M. Lauschke is CEO and shareholder of HepaPredict AB and a cofounder and shareholder of Shanghai Hepo Biotechnology Ltd. Magnus Ingelman-Sundberg is chairman of the board and shareholder of HepaPredict AB.
